# The Activity and Stability of p56Lck and TCR Signaling Do Not Depend on the Co-Chaperone Cdc37

**DOI:** 10.3390/ijms22010126

**Published:** 2020-12-24

**Authors:** Sarah Kowallik, Andreas Kritikos, Matthias Kästle, Christoph Thurm, Burkhart Schraven, Luca Simeoni

**Affiliations:** 1 Institute of Molecular and Clinical Immunology, Otto-von-Guericke University, 39120 Magdeburg, Germany; sarah.kowallik@med.ovgu.de (S.K.); perkizitore@gmail.com (A.K.); matthias.kaestle@med.ovgu.de (M.K.); christoph.thurm@med.ovgu.de (C.T.); burkhart.schraven@med.ovgu.de (B.S.); 2 Health Campus Immunology, Infectiology and Inflammation (GC-I^3^), Medical Faculty, Otto-von Guericke University, 39120 Magdeburg, Germany

**Keywords:** Cdc37, co-chaperone, Lck, tyrosine kinase, TCR signaling, heat shock protein 90 (Hsp90)

## Abstract

Lymphocyte-specific protein tyrosine kinase (Lck) is a pivotal tyrosine kinase involved in T cell receptor (TCR) signaling. Because of its importance, the activity of Lck is regulated at different levels including phosphorylation of tyrosine residues, protein–protein interactions, and localization. It has been proposed that the co-chaperone Cdc37, which assists the chaperone heat shock protein 90 (Hsp90) in the folding of client proteins, is also involved in the regulation of the activity/stability of Lck. Nevertheless, the available experimental data do not clearly support this conclusion. Thus, we assessed whether or not Cdc37 regulates Lck. We performed experiments in which the expression of Cdc37 was either augmented or suppressed in Jurkat T cells. The results of our experiments indicated that neither the overexpression nor the suppression of Cdc37 affected Lck stability and activity. Moreover, TCR signaling proceeded normally in T cells in which Cdc37 expression was either augmented or suppressed. Finally, we demonstrated that also under stress conditions Cdc37 was dispensable for the regulation of Lck activity/stability. In conclusion, our data do not support the idea that Lck is a Cdc37 client.

## 1. Introduction

The lymphocyte-specific protein tyrosine kinase p56Lck is one of the most well studied member of the Src-family kinases (SFKs), playing an essential role in the initiation of T cell receptor (TCR) signaling [1,2]. Lck possesses the typical structural organization of SFKs, including a SH4 domain for plasma membrane targeting, a unique domain required for the association with the co-receptors CD4 and CD8, a SH3 and a SH2 domain required for protein–protein interactions, a SH2-kinase domain linker, a kinase domain, and a negative regulatory tail [1,2,3]. The activity of Lck is regulated by two tyrosine residues, Y394 located in the activation loop within the kinase domain and Y505 lying in the negative regulatory tail. It is well established that phosphorylation on Y394 is mandatory to enzymatically activate Lck [4,5], whereas phosphorylation of Y505 inhibits Lck activation by closing Lck via an intramolecular interaction with its SH2 domain [6,7,8].

Upon TCR engagement by major histocompatibility complex (MHC) peptide ligands, Lck undergoes a conformational opening paralleling with de novo phosphorylation on Y394 which is crucial for the initiation of TCR signaling [4,5,9]. Subsequently, Lck phosphorylates tyrosine residues within the immunoreceptor tyrosine-based activation motifs (ITAMs), which are located in the intracellular tails of TCR-associated CD3 chains (for more comprehensive reviews, see [2,10,11]). This event allows the recruitment of the tyrosine kinase zeta chain of T cell receptor-associated protein kinase 70 (Zap-70) to the phosphorylated ITAMs, followed by Zap-70 phosphorylation and activation by Lck. Signaling is further propagated upon Zap-70-mediated phosphorylation of the transmembrane adaptor molecule linker for activation of T cells (LAT), thus culminating in T cell activation. The importance of Lck in T cell biology has been shown using different model systems. Initial studies using an Lck-deficient T cell line clearly showed that loss of Lck results in blunted TCR signaling [12]. Subsequent studies using peripheral murine T cells confirmed the pivotal role of Lck in TCR signaling [13]. Since TCR signaling regulates different aspects of T cells such as T cell development, activation, and homeostasis, it was hypothesized that loss of Lck would have a major impact on these biological processes. Indeed, Lck-deficient mice display a severe block of T cell development [14], impaired T cell activation [13,15], and defective T cell homeostasis [15].

The activity of Lck is tightly regulated by a number of sophisticated mechanisms [1,3,16]. In this regard, it was previously shown that the molecular chaperone heat shock protein 90 (Hsp90) is essential for membrane localization [17] and stability [17,18,19,20] of Lck. These studies also demonstrated that the active form of Lck is significantly more dependent on the chaperoning activity of Hsp90 than the catalytically inactive closed form of Lck [17,18,20]. Thus, the idea is that Hsp90 is an additional player in the regulation of Lck activity, which is required to maintain an active pool of Lck and to prevent its ubiquitin-mediated degradation.

Hsp90 is an abundant cytoplasmic molecular chaperone stabilizing and regulating the activity of a broad spectrum of client proteins (recently reviewed in [21]). The selectively of Hsp90 for its clients is determined by a set of co-chaperones, which bridge Hsp90 to the client protein. One of the most well characterized co-chaperons is Cdc37, which mediates the recruitment of protein kinases to Hsp90 [22,23,24]. Cdc37 interacts via its N-terminus with the client kinase, whereas the C-terminal region of Cdc37 binds to Hsp90 [25,26]. In addition to its scaffolding function, it has become evident that Cdc37 also has an important regulatory activity on the Hsp90 chaperone cycle [27,28,29].

A variety of kinases have been shown to be associated with Cdc37 [30], including Lck [31]. The importance of Cdc37 in the regulation of client kinase activity has been demonstrated by a number of studies in which the expression of Cdc37 was genetically manipulated. For example, it was shown that overexpression of Cdc37 increased the intracellular levels of the cleaved part of Ryk, a receptor tyrosine kinase-like Wnt-family member, and promotes its nuclear localization. In contrast, Cdc37 knockdown reduces its stability [32]. Similarly, overexpression of Cdc37 enhanced the expression of the protein kinase IRAK in 293T cells [33]. Using prostate cancer as a model, researchers found that overexpression of Cdc37 enhances c-Raf activity and increases CDK4 expression in human prostate epithelial cells [34]. Conversely, Cdc37 silencing was found to attenuate kinase signaling of the Raf-ERK and PI3K/protein kinase B (AKT) pathways and impair proliferation of Schwann cells [35]. Similarly, downregulation of Cdc37 in breast cancer cells paralleled with decreased levels of Akt [36]. Silencing of Cdc37 in human colon cancer cells reduced the expression of different kinases such as ErbB2, c-Raf, CDK4, and CDK6 and also impaired the activation of Akt [37]. Suppression of Cdc37 in HepG2 and Huh7 hepatoma cell lines enhanced CDK4 expression [38]. Finally, suppression of Cdc37 by RNA interference decreased the activity of Erk, Akt, and mTOR in carcinoma cells [39]. Collectively, these data clearly highlight the importance of Cdc37 in the regulation of the expression and/or the activity of different kinases.

Cdc37 has been shown to interact with Lck (although only in an in vitro cell-free system) [31] and it is also assumed that Cdc37 in complex with Hsp90 plays a role in the stabilization of Lck [20]. However, a direct demonstration of the role of Cdc37 in the regulation of Lck stability/activity is to date still missing. On the basis of these considerations, we decided to directly investigate whether Cdc37 is involved or not in the regulation of Lck. To this aim, we overexpressed Cdc37 or suppressed its expression in Jurkat T cells. Conversely to the studies mentioned above in which changes in the expression of Cdc37 altered the expression/activity of Cdc37 client kinases, we surprisingly found that the expression/activity of Lck was not affected by suppressing or increasing the expression of Cdc37. Moreover, Cdc37 did not appear to be involved in TCR signaling.

## 2. Results and Discussion

### 2.1. Overexpression of Cdc37 Did Not Affect Lck Expression, Lck Activation, and TCR Signaling

**Figure 1:**
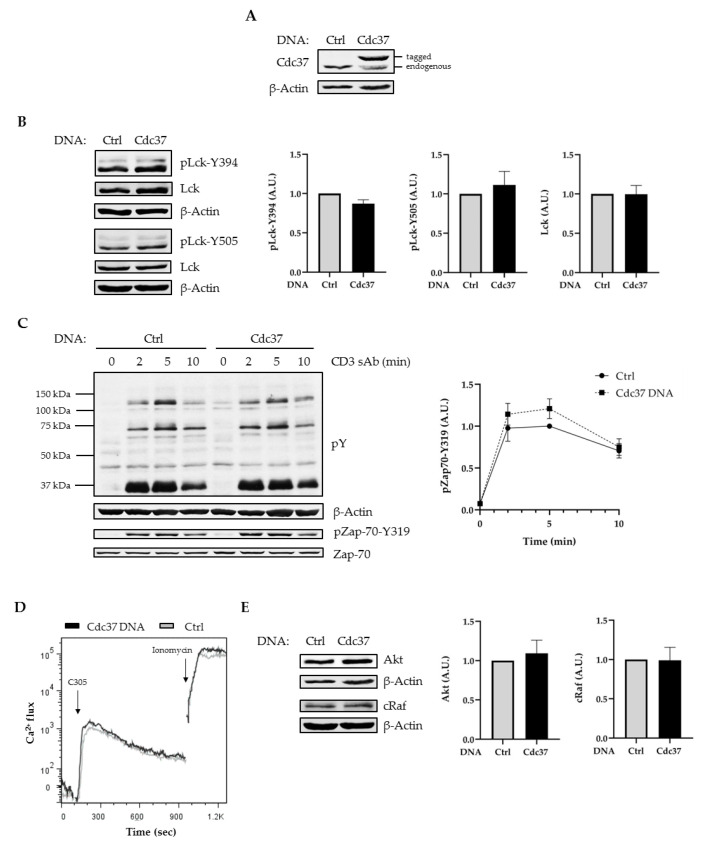
Cdc37 overexpression did not affect lymphocyte-specific protein tyrosine kinase (Lck) expression and T cell receptor (TCR) signaling. Jurkat T cells (JE6) were transfected with Ctrl or Cdc37 plasmids and cultured for 16 h. Cell lysates were analyzed by immunoblotting using the indicated Abs. (**A**) Immunoblot showing Cdc37 overexpression. (**B**) Immunoblots showing Lck expression and phosphorylation. Bands in (**B**) were quantified using the ImageStudio software and values were normalized to the corresponding total protein signal or β-actin signal. (**C**) After 16 h, cells were stimulated with soluble CD3 (sAbs) (clone UCHT1) for the indicated times. Subsequently, lysates were analyzed by immunoblotting using the indicated Abs. Bands in (**C**) were quantified as described in (**B**). The graph shows the phosphorylation level of zeta chain of T cell receptor-associated protein kinase 70 (Zap-70) as arbitrary units ± SEM (standard error of the mean) of four independent experiments. (**D**) JE6 cells were incubated with Indo-1AM, stimulated with the TCR clonotypic antibody C305, and Ca^2+^ flux was measured by flow cytometry. Ionomycin is used as positive control to induce maximum Ca^2+^ flux. (**E**) Immunoblots showing Akt and cRaf expression. Graphs show the expression levels of indicated molecules as arbitrary units ± SEM of three independent experiments. Significant *p*-values were calculated using the Student’s *t*-test.

To study the role of Cdc37 in the regulation of Lck, we initially performed overexpression experiments in Jurkat T cells (JE6). We used a construct coding for a tagged version of human Cdc37 to distinguish between the endogenous and the overexpressed form (Figure 1A). Then, 16 h after transfection, JE6 cells were lysed and the levels of total Lck as well as the levels of Lck phosphorylated on the regulatory sites, Y394 and Y505, were assessed by immunoblotting. Figure 1B shows that both the expression of total Lck and of its phosphorylated forms were not affected, despite significant increase in the expression of Cdc37. Thus, even though Hsp90 has been proposed to regulate the pool of constitutively active Lck [17,18,20], its co-chaperone appears to be dispensable in this process.

The activity of Lck is crucial for the induction of TCR-mediated signaling [12]. We next assessed whether global tyrosine phosphorylation, the phosphorylation of Zap-70, and calcium influx are altered upon Cdc37 overexpression. Figure 1C,D clearly shows that these signaling events were not altered in cells overexpressing Cdc37. These data additionally indicate that the overexpression of Cdc37 did not alter the stability of other kinases involved in TCR signaling. Indeed, the expression of Zap-70 (Figure 1C and [40]) as well as of Akt and Raf (Figure 1E) were not affected by the overexpression of Cdc37.

### 2.2. Suppression of Cdc37 by RNAi Did Not Affect Lck Expression, Lck Activation, and TCR Signaling

**Figure 2:**
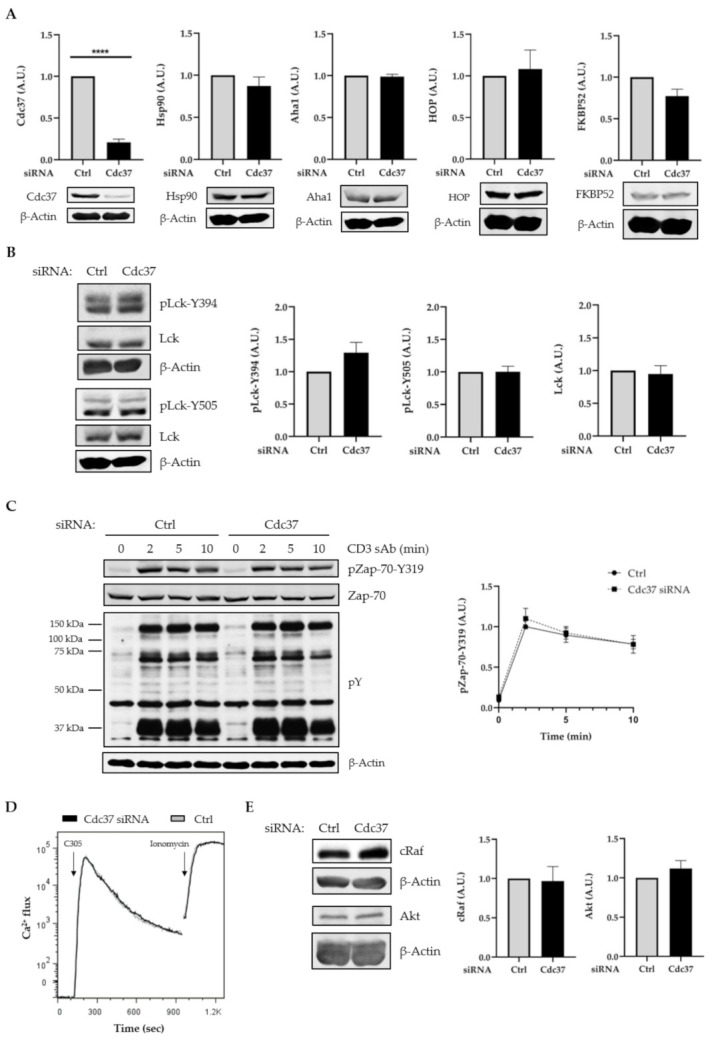
Cdc37 suppression did not impair Lck expression and TCR signaling. JE6 cells were transfected with Ctrl or Cdc37 siRNA and cultured for 48 h. Cell lysates were analyzed by immunoblotting using the indicated Abs. (**A**) Immunoblot showing Cdc37 suppression and the expression of components of the chaperone complex. Bands in (**A**) were quantified using the ImageStudio software and values were normalized to the corresponding β-actin signal. Graphs show the expression levels of the indicated molecules as arbitrary units ± SEM of three independent experiments. (**B**) Immunoblots showing Lck expression and phosphorylation. (**C**) 48 hours after transfection, cells were stimulated with a soluble CD3 antibody (sAb) (clone UCHT1) for the indicated times. Subsequently, lysates were analyzed by immunoblotting. Bands in (**C**) were quantified as in Figure 1. Graph shows the phosphorylation levels of Zap-70 as arbitrary units ± SEM of six independent experiments. (**D**) JE6 cells were incubated with Indo-1AM and stimulated with the TCR clonotypic antibody C305, and Ca^2+^ flux was measured by flow cytometry. Ionomycin was used to induce maximum Ca^2+^ flux. (**E**) Immunoblots showing Akt and cRaf expression. Graphs show the expression levels of indicated molecules as arbitrary units ± SEM of three independent experiments. Significant *p*-values were calculated using the Student’s *t*-test (****, *p* < 0.0001).

To further assess the function of Cdc37 in the regulation of Lck stability/activity, we next suppressed its expression by RNA interference (RNAi). To this aim, we used previously published siRNA duplex, which was shown to efficiently silence Cdc37 [37]. Jurkat T cells were transfected with Cdc37 siRNA or siRNA control. After 48 h, cells were harvested and assayed for Cdc37 expression. The data shown in Figure 2A demonstrate that Cdc37 was efficiently suppressed. Of note, downregulation of Cdc37 did not affect the expression of Hsp90 or the expression of the co-chaperones Aha-1, FKBP52, and Hop (Figure 2A). This observation is in line with previously published data, indicating that this siRNA does not alter the expression of other components of the chaperone complex [37]. In agreement with the overexpression data, we found that suppression of Cdc37 did not affect both the expression and the activation of Lck (Figure 2B). The kinases c-Raf [25,34,35,37] and Akt [35,36,37] are among the Cdc37 clients that have been described to depend on its co-chaperoning activity. Therefore, we assessed whether the expression of c-Raf and Akt was also affected in T cells in which Cdc37 was silenced. We surprisingly found that both c-Raf and Akt expression were not affected upon Cdc37 suppression (Figure 2E). This observation together with the overexpression data presented in Figure 1E indicate that c-Raf and Akt are likely not Cdc37 clients in T cells.

We next assessed the effect of Cdc37 suppression on TCR signaling. Again, also in this case, we did not see major differences when we analyzed global tyrosine phosphorylation, Zap-70 activation, and Ca^2+^ influx in cells in which the expression of Cdc37 was suppressed compared to control cells (Figure 2C,D). Taken together, these data strongly suggest that Cdc37 is dispensable for the regulation of the stability/activity not only of Lck but also for other kinases involved in TCR signaling.

### 2.3. Cdc37 Was Not Required for the Regulation of Lck under Stress Conditions

**Figure 3:**
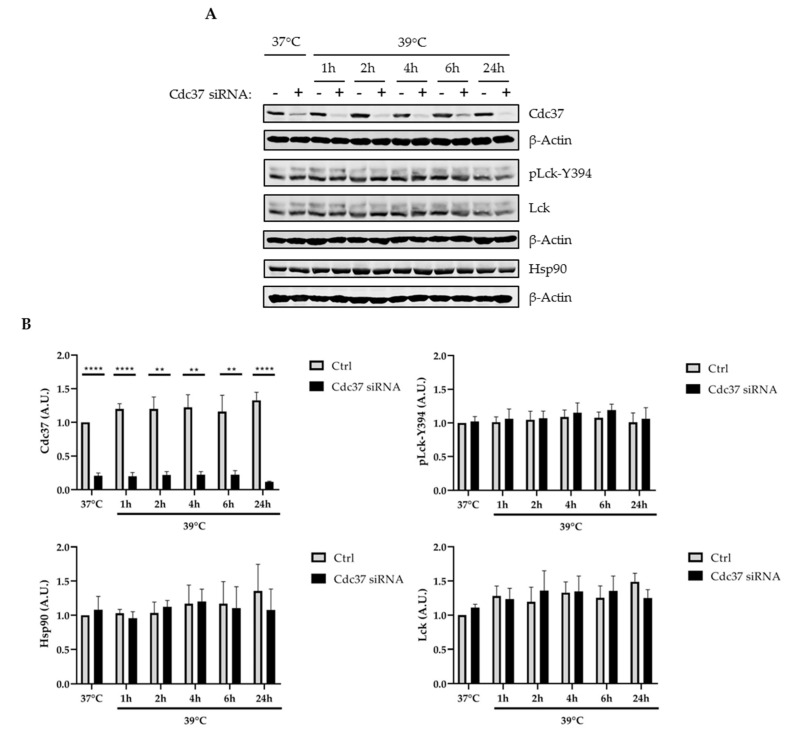
Cdc37 suppression did not influence Lck phosphorylation and expression under stress condition. JE6 cells were transfected with Ctrl (−) or Cdc37 (+) siRNA and cultured for 48 h. After 48 h, cells were incubated at 39 °C for indicated time points. Subsequently, cell lysates were analyzed by immunoblotting using the indicated antibodies. (**A**) Immunoblots showing the expression of the indicated molecules. Bands in (**A**) were quantified using the ImageStudio software and values of Lck-pY394 were normalized to the total Lck signal, whereas signals of the total proteins were normalized to the corresponding β-actin signal. (**B**) Graphs show the expression levels of the indicated molecules as arbitrary units ± SEM of four independent experiments. Significant *p*-values were calculated using the Student’s *t*-test (**, *p* < 0.01; ****, *p* < 0.0001).

The activity of chaperones is particularly important to prevent misfolding of proteins under stress conditions such as elevated temperatures [41,42]. To test whether the activity of Cdc37 is required for the stability of Lck under stress conditions, we cultured Jurkat T cells, in which Cdc37 was suppressed by RNAi, at 39 °C, and the expression of Lck and its phosphorylation status were assessed by Western blotting. The data in Figure 3 show that Cdc37 was also dispensable for the regulation of Lck under stress conditions.

### 2.4. Inhibition of Hsp90 Affected Lck Expression and Impaired TCR Signaling

**Figure 4:**
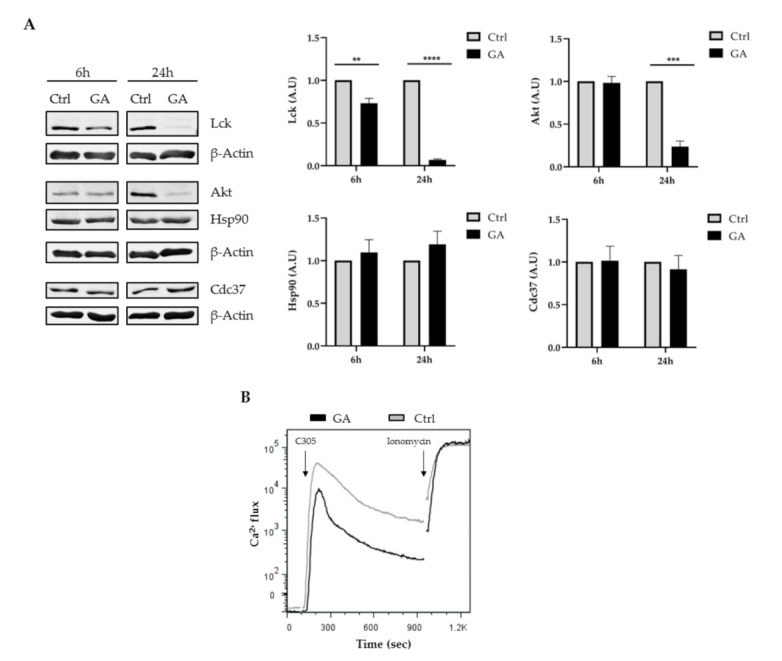
Inhibition of heat shock protein 90 (Hsp90) activity affected Lck expression and TCR signaling. JE6 cells were either left untreated (Ctrl) or treated with geldanamycin (GA) for 6 h or 24 h. Cell lysates were analyzed by immunoblotting using the indicated antibodies. (**A**) Immunoblot showing the expression of the indicated molecules. Bands in (**A**) were quantified using the ImageStudio software and values were normalized to the corresponding β-actin signal. Graphs show the expression levels of the indicated molecules as arbitrary units ± SEM of three independent experiments. (**B**) JE6 cells were either left untreated (Ctrl) or treated with geldanamycin for 6 h. Subsequently, JE6 cells were incubated with Indo-1AM and stimulated with the TCR clonotypic antibody C305, and Ca^2+^ flux was measured by flow cytometry. Ionomycin was used to induce maximum Ca^2+^ flux. Significat *p*-values were calculated using the Student’s *t*-test (**, *p* < 0.01; ***, *p* < 0.001; ****, *p* < 0.0001).

The data described above indicate that Cdc37 was dispensable for the regulation of Lck. To shed light onto the chaperoning mechanisms regulating Lck, we took advantage of a Hsp90 inhibitor. Geldanamycin (GA) is a benzoquinone ansamycin antibiotic that inhibits Hsp90 by blocking ATP binding [43]. Previous studies have shown that treatment of T cells with GA destabilizes the active form of Lck (i.e., phosphorylated on Y394) [17,18,20]. We indeed corroborated these observations, as treatment of Jurkat T cells with GA strongly reduced Lck expression (Figure 4A). The effect of GA was modest after 6 h incubation but very pronounced 24 h after GA addition. Similarly, we found that the expression of Akt, another client kinase of Hsp90, was also reduced upon incubation with GA (Figure 4A). Of note, GA did not affect the expression of both Hsp90 and Cdc37 (Figure 4A). To test the effects of GA on TCR signaling, we assessed Ca^2+^ influx using flow cytometry. In agreement with the effect of GA on Lck expression, Ca^2+^ influx was reduced upon GA treatment (Figure 4B). In conclusion, our data demonstrate that Hsp90 but not Cdc37 regulates Lck expression/activity and TCR signaling in Jurkat T cells.

## 3. Conclusions

Despite it having been proposed that Lck is a Cdc37 client, the data shown here do not support this hypothesis. It is possible that the activity/stability of Lck is regulated in concert by Cdc37 and other co-chaperones. Since Lck is an essential kinase in T cell biology, redundancy in the co-chaperoning system would be required to tightly regulate its expression. Alternatively, it is also possible that the chaperoning function of Hsp90 is not supported by Cdc37 but by other unknown co-chaperones to maintain Lck expression/activity and TCR signaling. Therefore, it will be important for the future to identify which co-chaperones are expressed in T cells to assists Hsp90 in the regulation of Lck expression and to maintain the proteostatic equilibrium necessary for TCR signaling.

## 4. Materials and Methods

### 4.1. Cell Culture

Jurkat T cells (JE6) were cultured in Roswell Park Memorial Institute (RPMI) 1640 medium (PAN Biotech, Aidenbach, Germany) with 10% (v/v) fetal bovine serum (FBS; Biowest, Nuaillé, France).

### 4.2. Overexpression of Cdc37

For overexpression, Cdc37 plasmid (NM_007065, Origene, Rockville, MD, USA) and a control plasmid (pEF-Bos) were used. DNA electroporation was performed with a Gene Pulser II System (BIORAD, Hercules, CA, USA). We resuspended 2 × 10^7^ Jurkat T cells in 350 μL RPMI/10% FBS and transferred the mixture with 30 μg of either control or Cdc37-expressing plasmids into a 4 mm electroporation cuvette. Electroporations were performed with 230 V and 950 μF. Subsequently, cells were transferred in 50 mL RPMI/10% FBS (25 mL fresh/25 mL conditioned) and cultured for 16–20 h.

### 4.3. Suppression of Cdc37

For the downregulation of Cdc37, we synthesized siRNA duplex containing 19 nucleotides with 2 thymidine 3′ overhangs from Invitrogen (Invitrogen, Carlsbad, CA, USA). The following sequences were used: for Cdc37: 5′-UUC CAC GAA GGU CUU GUG U-3′ [37], and as negative control, we used a Renilla luciferase siRNA duplex: 5′-UUG AUC CUA CAU UAC UUG G-3′ [44]. siRNA duplex electroporation was performed as previously described [44]. We transfected 2 × 10^7^ JE6 cells with 200μM of either Renilla or Cdc37 siRNA duplex and cultured the mixture for 48 h in 50 mL RPMI/10% FBS (25 mL fresh/25 mL conditioned).

### 4.4. Cell Stimulation and Immunoblot Analysis

For cell stimulation, Jurkat T cells were treated at 37 °C with 2μg/mL soluble anti-CD3 (clone UCHT1, eBioscience, San Diego, CA, USA) for the indicated time points. Cells were lysed in lysis buffer containing 1% Nonidet P-40 (NP-40), 1% laurylmaltoside (*N*-dodecyl β-d-maltoside), 50 mM Tris (pH 7.5), 140 mM NaCl, 10 mM ethylenediaminetetraacetic acid (EDTA), 10 mM NaF, 1 mM phenylmethylsulfonyl fluoride, and 1 mM Na_3_VO_4_. For immunoblot analyses, proteins were separated by sodium dodecyl sulfate polyacrylamide gel electrophoresis, transferred onto nitrocellulose membrane, and blotted with the following antibodies: anti-pY^394^ Lck (Cell Signaling Technology, Danvers, MA, USA), anti-pY^505^ Lck (Cell Signaling Technology, Danvers, MA, USA), anti-pY^319^ Zap70 (Cell Signaling Technology, Danvers, MA, USA), anti-p-Tyr (Biomol GmbH, Hamburg, Germany), anti-Lck (Santa Cruz Biotechnology, Dallas, TX, USA), anti-Zap70 (Santa Cruz Biotechnology, Dallas, TX, USA), anti-Akt (Cell Signaling Technology, Danvers, MA, USA), anti-c-Raf (Cell Signaling Technology, Danvers, MA, USA), anti-β-actin (Sigma-Aldrich, Munich, Germany), anti-Cdc37 (Santa Cruz Biotechnology, Dallas, TX, USA), anti-Hsp90 (Cell Signaling Technology, Danvers, MA, USA), anti-Aha1 (Santa Cruz Biotechnology, Dallas, TX, USA), anti-HOP (Santa Cruz Biotechnology, Dallas, TX, USA), and anti-FKBP52 (Bethyl Laboratories, Montgomery, TX, USA). For protein detection, membranes were incubated with labelled secondary antibodies and signals were analyzed using the Odyssey system (LI-COR, Lincoln, NE, USA).

### 4.5. Geldanamycin Treatment

Cells were incubated with 5 μM geldanamycin (GA) (Sigma-Aldrich, Munich, Germany) for 6 h or 24 h at 37 °C and lysed immediately, as described above.

### 4.6. Ca^2+^ Influx Measurement

To measure Ca^2+^ flux, we transfected cells with Cdc37 plasmids or Cdc37 siRNA, as described above, and incubated with 4 μM Indo-1 AM (Invitrogen, Carlsbad, CA, USA) at 37 °C for 90 min. Cells were washed, resuspended in RPMI without phenol red/10% FBS (Biochrom, Cambridge, UK), and stimulated with C305 (clonotypic TCR antibody). Ca^2+^ flux was measured on a LSRII flow cytometer (BD Biosciences, San Jose, CA, USA). Ionomycin was added to the samples at the end of the measurement as a positive control to measure maximum Ca^2+^ flux. Raw data were transferred to FlowJo V.7.8.5 (Tree Star, Ashland, OR, USA) for the analysis.

### 4.7. Statistics

Statistical analyses were performed using GraphPad Prism (GraphPad Software Inc., San Diego, CA, USA). All data are presented as mean ± SEM (standard error of the mean). *p*-values were determined by an unpaired two-tailed Student’s *t*-test.

## Data Availability

The data presented in this study are available on request from the corresponding author.

## Abbreviations

Akt	Protein kinase B
GA	Geldanaymycin
FBS	Fetal bovine serum
Hsp90	Heat shock protein 90
ITAM	Immunoreceptor tyrosine-based activation motif
Lck	Lymphocyte-specific protein tyrosine kinase
MHC	Major histocompatibility complex
RNAi	RNA interference
TCR	T cell receptor
Y	Tyrosine
Zap-70	Zeta chain of T cell receptor-associated protein kinase 70
